# Increased Catecholamine Levels and Inflammatory Mediators Alter Barrier Properties of Brain Microvascular Endothelial Cells *in vitro*

**DOI:** 10.3389/fcvm.2020.00073

**Published:** 2020-05-05

**Authors:** Cora Ittner, Malgorzata Burek, Stefan Störk, Michiaki Nagai, Carola Y. Förster

**Affiliations:** ^1^Department of Anaesthesia and Critical Care, University of Würzburg, Würzburg, Germany; ^2^Comprehensive Heart Failure Center, University of Würzburg, Würzburg, Germany; ^3^Department of Internal Medicine, General Medicine and Cardiology, Hiroshima City Asa Hospital, Hiroshima, Japan

**Keywords:** blood-brain barrier, microvascular endothelium, cEND cell line, takotsubo syndrome, catecholamines, inflammation, *in vitro*

## Abstract

Recent studies have suggested a pathogenetic link between ischemic stroke and Takotsubo cardiomyopathy (TCM) with poor outcome, when occurring simultaneously. Increased catecholamine (CAT) levels as well as elevated inflammatory mediators (INF) are found in the blood of patients with ischemic stroke concomitant with Takotsubo syndrome (TTS). On molecular level, the impact of these stressors combined with hypoxemia could compromise the integrity of the blood brain barrier (BBB) resulting in poor outcomes. As a first step in the direction of investigating possible molecular mechanisms, an *in vitro* model of the described pathological constellation was designed. An immortalized murine microvascular endothelial cell line from the cerebral cortex (cEND) was used as an established *in vitro* model of the BBB. cEND cells were treated with supraphysiological concentrations of CAT (dopamine, norepinephrine, epinephrine) and INF (TNF-α and Interleukin-6). Simultaneously, cells were exposed to oxygen glucose deprivation (OGD) as an established *in vitro* model of ischemic stroke with/without subsequent reoxygenation. We investigated the impact on cell morphology and cell number by immunofluorescence staining. Furthermore, alterations of selected tight and adherens junction proteins forming paracellular barrier as well as integrins mediating cell-matrix adhesion were determined by RT-PCR and/or Western Blot technique. Especially by choosing this wide range of targets, we give a detailed overview of molecular changes leading to compromised barrier properties. Our data show that the proteins forming the BBB and the cell count are clearly influenced by CAT and INF applied under OGD conditions. Most of the investigated proteins are downregulated, so a negative impact on barrier integrity can be assumed. The structures affected by treatment with CAT and INF are potential targets for future therapies in ischemic stroke and TTS.

## Introduction

Recent clinical publications and literature reviews point out that takotsubo syndrome (TTS) occurring in combination with ischemic stroke is a notable clinical constellation (1). The link between these two pathologies is not well-understood yet, especially not on molecular level.

Takotsubo cardiomyopathy (TCM) is a cardiac pathology at first glance mimicking acute coronary syndrome (ACS). The most important clinical feature is the absence of coronary occlusion which differentiates TCM from ACS (2). Characteristic wall motion abnormalities predominantly affecting the left ventricle are observed. This phenomenon is described as apical ballooning. Patients are mostly postmenopausal women, who present dyspnea, chest pain as well as new ECG alterations and/or elevated cardiac enzymes (2). Precursory cerebrovascular disease no longer interdicts to diagnose TCM. In one to two thirds of patients suffering from TCM, a previous psychically or physically stressful trigger can be identified (3, 4).

Interestingly, there is no strict sequence, whether ischemic stroke or TCM develop first. Ischemic stroke is described as a neurological precipitant of TTS. In contrast, thrombus formation caused by left ventricular dysfunction within TCM can also lead to ischemic stroke. Hereby, ischemic strokes in patients with TTS are usually severe and classified by an initially higher NIHSS score in comparison with patients without TTS. Poor outcomes are frequent in the patients of ischemic stroke with TTS (1, 5).

Patients presenting the described pathological pattern, show characteristically altered laboratory findings: Supraphysiological CAT blood levels can be determined (6). This reflects the plausible theory of TTS pathogenesis consisting in raised CAT, which are assumed to mediate myocardial wall motion abnormalities (3). Furthermore, elevated inflammatory markers are found (1). This correlates with the known fact of raised INF in ischemic areas after stroke. Resident cerebral immune cells as well as migrated immune cells synthesize, inter alia, pro-inflammatory cytokines like TNF-α and IL-6 (7). Especially cardioembolic strokes are associated with increased CRP, TNF-α, and IL-6 levels compared to other subtypes of ischemic insults (8). Although, the role of the two mentioned cytokines is still diversely discussed, clinical and experimental data suggest an aggravation of ischemic brain damage by inflammatory spillover. Furthermore, dysfunction of the cerebral microvascular endothelium in the frame of ischemic stroke is promoted by systemic inflammation (7).

The depicted clinical situation presents a pathological process within the brain-heart axis. In general, the brain-heart axis is a dual system, which works up and down stream: cardiac dysfunction may trigger neurovascular deterioration and vice versa (9). Ischemic stroke and TCM seem to interact due to the influence of the insular cortex on the modulation of the autonomic nervous system. *In vivo* animal models as well as human studies have shown ischemic lesions affecting the insular cortex cause autonomic dysregulation (10), especially when concerning the left hemisphere, the impairment of the insular cortex produces a sympathetic overdrive reflected by elevated CAT levels (11). Clinical findings correspondingly suggest a predominance of ischemic strokes affecting the insular cortex and/or adjacent brain regions preceding TTS (12).

As part of the brain-heart axis, the BBB reacts to hypoxic events caused by cardiac impairment and vice versa (9). Therefore, when investigating pathologies of the brain-heart axis the BBB has to be included in speculation on possible pathogenetic mechanisms.

Sustained integrity of the BBB is mandatory for maintaining the homeostasis of the central nervous system (CNS). Thus, the CNS gets its unique position by not being exposed unselectively to systemic alterations such as inflammation (13). Restricted permeability is provided by microvascular endothelial cells forming dense cell-cell contacts: a dynamic interface is ensured by tight junctions (TJ) and adherens junctions (AJ) building a restrictive barrier (14, 15). If circulating noxious agents affect vascular endothelium itself, TJ as well as AJ could not be maintained properly leading to a breakdown of the BBB (16). Cerebral vascular endothelium forming the BBB, on its blood facing side, is constantly exposed to systemically circulating INF, and elevated CAT blood levels. The proinflammatory cytokines TNF-α and IL-6, as elevated in acute TTS (1) are proven to be harmful factors for the integrity of the BBB by compromising TJ protein as well as AJ protein expression *in vitro* (17, 18). Systemical administration of epinephrine lead to a disruption of the BBB *in vivo* (19). Furthermore, focal ischemia results in a breakdown of the BBB, which produces increased permeability. Aggravation of brain damage by secondary effects such as brain edema is provoked (13). TJ proteins claudin-5, occludin as well as AJ protein VE-cadherin were shown to be targets of hypoxic conditons *in vitro* (20).

Based on these assumptions, we suggest that the severity of ischemic stroke in patients with TTS is caused by aggravated dysfunction of the BBB. An intensified impairment of the vascular endothelial cells generated by elevated CAT levels and INF combined with hypoxia is hypothesized. As a first step of investigating this pathologic constellation, an *in vitro* experiment was designed. Supraphysiological CAT levels combined with INF were applied under OGD (20). An immortalized murine cerebral endothelial cell line (cEND) was chosen as a well-established *in vitro* model of the BBB (21). The present study provides a comprehensive overview of molecular changes of cerebral microvascular endothelium, exposed to an *in vitro* simulation of characteristic laboratory findings in patients suffering from TTS and ischemic stroke simultaneously.

## Materials and Methods

### Chemicals

TNF-α, Dopamine, Epinephrine as well as Norepinephrine were purchased from Sigma-Aldrich. Interleukin-6 was purchased from Thermo Fisher. Epinephrine and Norepinephrine were dissolved in 0.5 M hydrochloric acid, Interleukin-6 in 100 nM acetic acid. TNF-α and Dopamine are readily soluble in water, so cell culture medium was used for dissolving. Final dilution of every agent was made shortly before the experiment in cell culture medium.

### Cell Culture

As an *in vitro* model of the Blood Brain Barrier (BBB) we used the murine microvascular cerebral endothelial cell line cEND. Cells were isolated, immortalized, and cultivated as described previously (21–23).

### Cell Treatment

Cells were grown to confluence within 5 days in Dulbeccos's modified eagles medium (DMEM) growth medium on 6-well-plates coated with gelatin. Growth medium consisted of DMEM (Sigma-Aldrich, D5796) containing 10% heat-inactivated fetal calf serum (FCS), 50 U/ml penicillin-streptomycin, 2% L-glutamine, 2% MEM vitamins, 2% non-essential amino acid solution (NEAA), and 2% sodium pyruvate. Subsequently, cells were differentiated for 48 h with DMEM differentiation medium containing 1% FCS as well as 50 U/ml penicillin-streptomycin. Stress factors were applied under different incubation conditions. Stress factors were defined as a combination of CAT [150 μM dopamine (24), 1 μM epinephrine (25), 1 μM norepinephrine (25)], and INF [100 nM TNF-α (18) and 5 pg/ml Interleukin-6 (26)]. For application on cells, stress factors were diluted to final concentration in DMEM differentiation medium or DMEM medium without glucose. Solvent of each agent was added in same concentration to control.

### Oxygen Glucose Deprivation (OGD) and Reoxygenation

After differentiation cells were washed twice with PBS. Subsequently, 1.5 ml DMEM without glucose per well were added, followed by incubation under OGD condition for 4 h. OGD condition was defined as 1% O_2_, 5% CO_2_, 37°C and saturated humidity atmosphere as previously described (20). Control was incubated simultaneously under normoxia condition in 1.5 ml DMEM medium containing glucose per well. After OGD, samples were analyzed directly or reoxygenated for 20 h. Reoxygenation was conducted by washing cells twice with PBS, adding 3 ml DMEM medium containing glucose per well and incubation under normoxia condition (5% CO_2_, 37°C, saturated humidity atmosphere) (20, 27).

### Immunofluorescence Staining

Cells were cultivated on collagen IV-coated coverslips (diameter of 12 mm). Cultivation was conducted as described above. After finished treatment, cells were washed twice with PBS. Fixation for 10 min, with ice-cold methanol, followed by washing (twice; PBS), and 15 min, of PBS containing 1% BSA. Before incubation with primary antibodies overnight at 4°C, samples were blocked for 1 h at RT with PBS containing 1% BSA and 5% normal swine serum. As primary antibodies were chosen: Claudin-5 (1:500; Thermo Fisher (Alexa fluor), #352588) and ZO-1 (1:500; Thermo Fisher, #402300) diluted in PBS with 1% BSA/ 5% normal swine serum. After washing (3 times for 5 min. with PBS), secondary antibodies diluted in PBS with 1% BSA/ 5% normal swine serum were applied: DAPI (1:3000; Sigma-Aldrich, #D9542) and Alexa fluor 594 donkey anti rabbit IgG (1:2000; Thermo Fisher, #A21207). Coverslips were fixed onto slides by using Mountant permaflour (Thermo Fisher, #TA-030-FM) and dried overnight at RT. Subsequently, analysis was performed with a Keyence fluorescence microscope (BIOREVO BZ-9000). The number of cells with intact DAPI-stained nuclei was estimated by counting the nuclei in a defined area of interest for each treatment.

### Western Blot Analysis

Western Blot analysis were performed as previously described (28). Briefly, cells were washed twice with ice cold PBS before being lysed in RIPA buffer containing protease inhibitors cocktail (Roche) and harvested. After sonication (10 times for 0.5 sec on ice), samples were centrifuged (11,000 rcf for 1 min) and supernatant was saved for quantification of protein content. Protein content was determined by BCA protein Assay Kit (Thermo Fisher). Protein separation was conducted using NuPAGE Kit (Thermo Fisher). Previously, samples were mixed with Laemmli buffer and reducing agent as indicated, followed by denaturation of 10 min. at 70°C. A NuPage 4–12% Bis-Tris-Gel (Thermo Fisher) was used for separation. The blot to a PVDF membrane was accomplished by electrophoretically wet transfer overnight (4°C) using a Electrophoretic Transfer Cell (BioRad). Subsequently, membrane was washed in PBS-T (3 times for 10 min.), blocked with 5% non-fat dry milk in PBS (1 h) and incubated overnight (4°C) with the primary antibody diluted in PBS containing 1% BSA. As primary antibodies we used: mouse anti-claudin-5 (1:500; Thermo Fisher, #35-2500), guinea pig anti-occludin (1:200; Acris, #358-504), rabbit anti-ZO-1 [1:500; Thermo Fisher, #40-2300), rat anti-VE-cadherin [pure; generated with hybridoma technique (29)] and anti-ß-aktin-HRP (1:25.000; Sigma-Aldrich, #A3854). Before applying secondary antibodies, membrane was washed with PBS-T (PBS with addition of 0.1% Tween) (3 times for 10 min) and blocked with 5% non-fat dry milk in PBS (20 min). Secondary antibodies were: anti-mouse IgG (1:3000; Roche, #12015218001), anti-guinea pig IgG (1:5000; Santa Cruz Biotechnology, #sc2438), anti-rabbit IgG (1:3000; Cell Signaling Technology, #7074S), and anti-rat IgG (1:5000; Thermo Fisher, Cat#61-9520). After washing the membrane with PBS-T (3 times for 10 min), incubation in ECL solution (2 min) followed. For imaging, Fluorchem FC2 MultiImager II (AlphaInnotech) was used. Density of protein bands was determined by ImageJ software (version 1.52a).

### Real-Time PCR

Real-time PCR analysis were performed as previously described (30). Briefly, cells were washed twice with sterile PBS. Subsequently, samples were harvested and RNA was isolated as well as purified from lysed cells following manufacterer's instructions of Nucleospin RNA (Macherey-Nagel, #740955). Reverse transcription for generating cDNA was performed by using High Capacity cDNA Reverse Transcription Kit (Applied Biosystems, #4368814). TaqMan Fast Advanced Master Mix (Applied Biosystems, #4444557) with 1 μg cDNA for RT-PCR. For determining gene expression, the following TagMan Gene Expression Assay primers were chosen: Claudin-5 (Mm00727012_s1), occludin (Mm00500912_m1), ZO-1 (Mm00493699_m1), VE-cadherin (Mm00486938_m1), Itgα1 (Mm01306375_m1), Itgαv (Mm00434486_m1). All purchased from Thermo Fisher. Calnexin RNA was used as the housekeeping gene (Canx; Mm00500330_m1). Subsequently, reverse transcription and amplification was conducted by using 7300 Real Time PCR System (Applied Biosystems) and under the following conditions: 95°C for 10 min, 50 cycles for each target, 60°C for 1 min.

### Analysis and Statistics

Data are presented as mean ± standard error of the mean (SEM). Number of independent experiments is indicated under figures. Statistical significance was evaluated by One-way ANOVA with Tukey's correction for multiple comparison using GraphPad Prism. Statistical significance was assumed for *P* < 0.05.

## Results

We wanted to examine, whether supraphysiological CAT concentrations and INF, as found in TTS patients (1), change AJ and TJ structure under normoxic and ischemic conditions. We chose VE-cadherin as a representative of AJ proteins and claudin-5, ZO-1 as well as occludin as representative of TJ proteins. In addition, integrin-α-1, and integrin-α-v were selected as targets reflecting the possible interaction with extracellular matrix (ECM) components. Integrin-α-1, a subunit of the collagen receptor integrin α1β1, binds collagen type IV (31), whereas integrin-α-v forms part of the vitronectin receptor αvβ3 (32). We used cEND cells, an immortalized murine microvascular endothelial cell line from the cerebral cortex, which is a well-established *in vitro* model of the BBB (21).

### Barrier Compromising Effects of Catecholamines and Inflammatory Mediators on cEND Cells Under Normoxic Conditions

**Figure 1:**
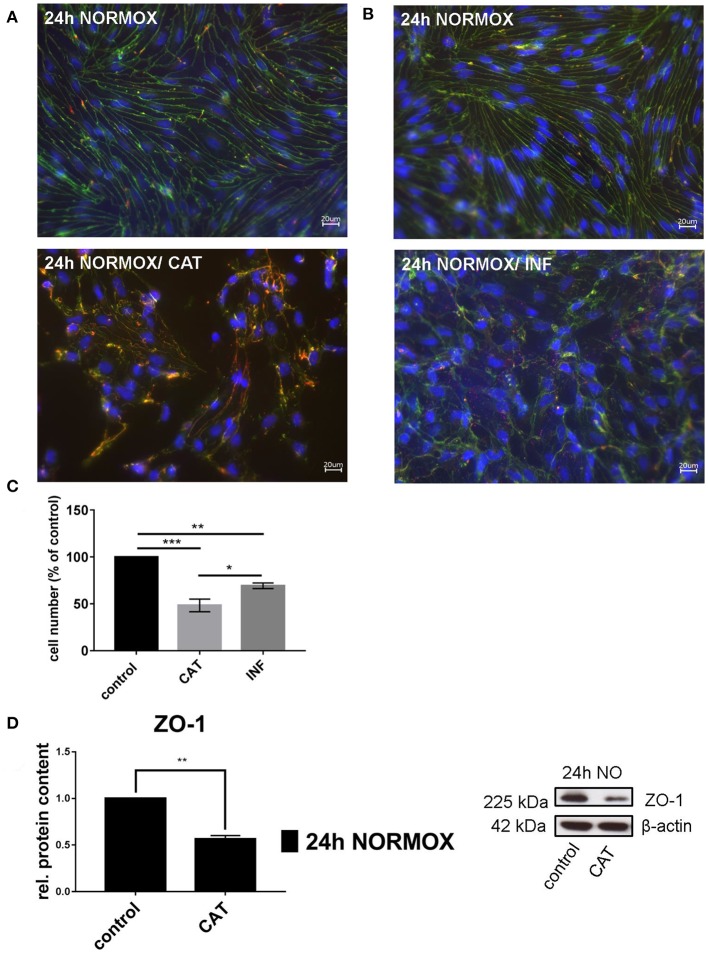
Effects of catecholamines and inflammatory mediators under normoxia conditions on cEND cells. Cells were grown to confluence with subsequent differentiation. Catecholamines (CAT) or inflammatory mediators (INF) were applied for 24 h under normoxia conditions (24 h NORMOX). **(A,B)** Immunofluorescence staining of tight junction proteins claudin-5 (green) and ZO-1 (red) as markers of morphological changes of the endothelial cell monolayer. DAPI (blue) was used for staining of nuclei. Magnification 400 times, scale bar 20 μm. **(C)** Cell number after CAT and INF treatment normalized to control. **(D)** CAT induced changes of ZO-1 protein expression in cEND cells investigated by Western blot. Data is presented as the means (± SEM) of *n* = 5 independent experiments. Altered protein expression was normalized to β-actin. ZO-1 protein level changes are expressed as fold over untreated control, which was set 1. *P < 0.05, **P < 0.01, ***P < 0.001.

To investigate how CAT or INF affect cEND cells, we treated cEND cells for 24 h under standard culturing conditions, called normoxia conditions (24 h NORMOX). We used a mixture of 150 μM dopamine (24), 1 μM epinephrine (25), and 1 μM norepinephrine (25) for CAT samples. Treatment with INF consisted of 100 nM TNF-α (18) and 5 pg/ml Interleukin-6 (26). All stressors were diluted in differentiation medium (1% FCS) to final concentration. Potential effects on cell morphology were determined by staining of cell-cell junctions using anti-claudin-5 and ZO-1 antibodies (Figure 1). As observed in control samples, the expressed claudin-5 and ZO-1 were located at the cell-cell contacts (Figures 1A,B) (21). After treatment with CAT or INF, the cell morphology was altered and the localization of junctional proteins at cell-cell junction sites was reduced. Treatment with CAT resulted in a disruption of the homogenous cell monolayer, while the cellular morphology was still spindle-shaped (Figure 1A). The percentage of cells with intact nuclei was significantly lower in samples treated with CAT compared to the control (Figure 1C). cEND cells lost their elongated spindle-shaped morphology (21) due to treatment with INF and showed more cobble-stone-like phenotype (Figure 1B). The number of cells with intact nuclei was also significantly lower than in the untreated control (Figure 1C). As demonstrated in Figure 1C, CAT alone caused a stronger breakdown of the homogenous cell monolayer compared to INF. A lower number of cells with intact nuclei indicates that CAT and INF treatment induces cell death in cEND cells.

To investigate whether the barrier properties of cEND cells are also affected at the protein and mRNA, cells were treated as described above. Subsequently, RT-PCR and Western Blot were performed (Figure 1, Table 1). Protein level of ZO-1 was reduced by 0.57 ± 0.04-fold while claudin-5, occludin and VE-cadherin were not altered by the administration of CAT (Figure 1D). CAT caused a decreased gene expression of claudin-5 to 0.47 ± 0.02-fold, ZO-1 to 0.38 ± 0.05-fold, and VE-cadherin to 0.55 ± 0.02-fold of control. A change in the mRNA level of occludin could not be detected. Integrin-α-1 mRNA was decreased by 0.19 ± 0.02-fold while integrin-α-v expression increased up to 1.82 ± 0.24-fold of control. Protein expression of selected targets was not significantly altered by INF (results not shown). INF led to impaired gene expression of occludin (0.39 ± 0.05-fold), ZO-1 (0.51 ± 0.05-fold), and VE-cadherin (0.85 ± 0.02-fold), but did not alter claudin-5 mRNA level. Integrin-α-v gene expression increased up to 3.31 ± 0.54-fold after INF treatment, while no differences were detected in integrin-α-1 mRNA level (Table 1).

**Table 1:** Modulated target gene expression in cEND cells by elevated catecholamine levels or inflammatory mediators under normoxia conditions for 24 h.

	**Claudin-5**	**ZO-1**	**Occludin**	**VE-cadherin**	**Itg α 1**	**Itg α v**
CAT	0.47 ± 0.02***	0.38 ± 0.05***	1.05 ± 0.11n.s.	0.55 ± 0.02***	0.19 ± 0.02***	1.82 ± 0.24*
INF	1.18 ± 0.21n.s.	0.51 ± 0.05***	0.39 ± 0.05***	0.85 ± 0.02**	0.68 ± 0.15n.s.	3.31 ± 0.54**

Catecholamines (CAT) or inflammatory mediators (INF) were applied for 24 h under normoxia conditions. After total RNA-extraction from lysed cells, mRNA was quantified by real-time PCR. Fold expression vs. untreated control, which was set 1. Values are the means (± SEM) of n = 5 (INF) or n = 6 (CAT) independent experiments, followed by statistical significance *P < 0.05, **P < 0.01, ***P < 0.001. n.s, not significant.

### Barrier Compromising Effects of Catecholamine Levels and Inflammatory Mediators on cEND Cells in Hypoxic Condition *In vitro*

We combined elevated levels of CAT and INF with hypoxic and hypoglycemic conditions called oxygen glucose deprivation (OGD) with or without subsequent reoxygenation. OGD was previously established in cEND as an *in vitro* model of ischemic stroke (20). From that point on, CAT and INF were combined and labeled as stress factors. Stress factors were diluted to final concentration in differentiation or glucose-free medium to be used under selected incubation conditions. Four different incubation conditions were defined: 4 h of normoxia (4 h NORMOX), 24 h of normoxia (24 h NORMOX), 4 h of OGD, and 4 h of OGD with subsequent reoxygenation under normoxia condition (REOX).

**Figure 2:**
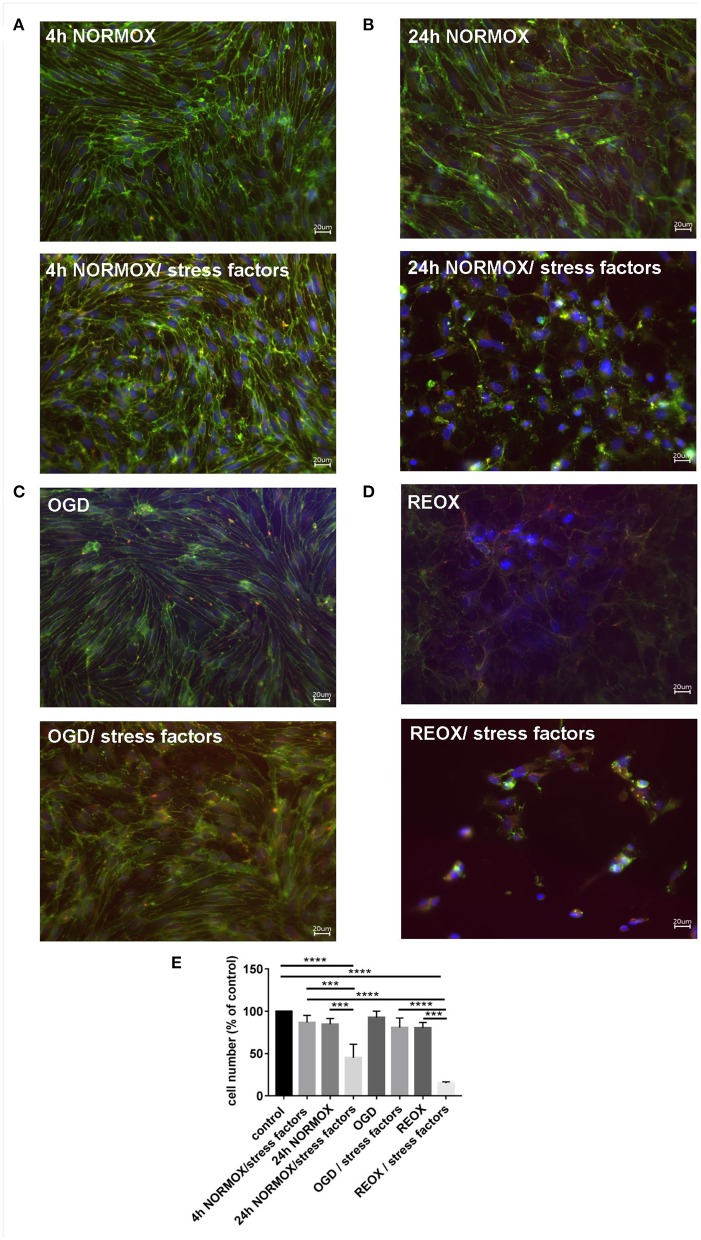
Loss of morphological integrity of cEND cells exposed to elevated catecholamine levels and inflammatory mediators. Immunofluorescence staining of tight junction proteins claudin-5 (green) and ZO-1 (red) as markers of morphological changes of the endothelial cell monolayer. DAPI (blue) was used for staining nuclei. Cells were grown on cover slips to confluence. After differentiation cEND cells were treated with combination of catecholamines and inflammatory mediators (stress factors). Stress factors were administered under different incubation conditions. Magnification 400 times, scale bar 20 μm. **(A)** Stress factor application for 4h under normoxia conditions (4 h NORMOX). **(B)** Stress factor application for 24 h under normoxia conditions (24h NORMOX). **(C)**. Stress factor application for 4 h under oxygen glucose deprivation conditions (OGD). **(D)** Stress factor application for 4 h under OGD conditions with 20 h of subsequent reoxygenation under normoxia conditions (REOX). **(E)** Cell number in treatments shown in **(A–D)** normalized to control. ***P < 0.001, ****P < 0.0001.

First, we analyzed the cell morphology after these treatments. The cells were grown on coverslips, differentiated, and incubated as described above (Figure 2). Possible effects on cell morphology were determined by staining of TJs with anti-claudin-5 and -ZO-1 antibodies. Cells grown without stress factors under normoxia conditions (4 h NORMOX and 24 h NORMOX) as well as OGD condition showed no changes in the cell morphology (Figures 2A–C) nor in cell number (Figure 2E). cEND cells showed a homogenous endothelial monolayer and the typical elongated spindle-shaped morphology as described before (21, 22) Both stained TJ proteins, claudin-5, and ZO-1, were located at the TJs (Figures 2A–C). The reoxygenation sample without stress factors showed a disrupted cell monolayer with irregular cellular morphology and delocalized TJ-proteins (Figure 2D) but no reduced cell number (Figure 2E). The effects of stress factors applied under normoxia conditions depended on time. After 4 h of treatment, endothelial cell monolayer changed slightly, while after 24 h of treatment, stress factors led to complete destruction of the cell monolayer (Figures 2A,B/stress factors) and a significant reduction of cell number (Figure 2E). Combination of OGD with stress factors potentiated these effects (Figures 2C/stress factors and Figure 2E). Morphologically, the most severe effect was caused by the administration of stress factors under reoxygenation conditions (Figure 2D/stress factors and Figure 2E). A typical morphological phenotype of cEND cells was no longer observed. The cells completely lost their spindle-shaped appearance and showed an irregular morphology without monolayer formation and significantly reduced cell numbers. Stained TJ proteins were visible as small aggregates outside the cell-cell contacts (Figure 2D/stress factors).

### Elevated Catecholamine Levels and Inflammatory Mediators Combined With Oxygen Glucose Deprivation Cause a Change in Gene Expression

We performed a real-time PCR, in order to investigate how the treatment with stress factors under different incubation conditions influences the gene expression of selected targets in cEND cells. First, we compared effects of stress factors under 4 h NORMOX, OGD, and REOX conditions, respectively (Table 2). Claudin-5 (0.49 ± 0.07-fold) and ZO-1 (0.7 ± 0.08-fold) gene expression was downregulated by OGD alone but upregulated again after REOX (claudin-5 0.88 ± 0.07-fold; ZO-1 1.26 ± 0.06-fold). This upregulation was not observed in combination with stress factor treatment. Similar changes were observed for occludin mRNA. VE-cadherin mRNA was significantly increased by OGD alone up to 1.56 ± 0.11-fold, followed by a decrease after REOX to 1.02 ± 0.03-fold. REOX in combination with stress factors caused a downregulation to 0.41 ± 0.06-fold of control. Integrin-α-1 was induced by REOX alone up to 1.43 ± 0.12-fold, but reduced to 0.05 ± 0.06-fold after REOX in combination with stress factors. Integrin-α-v mRNA was upregulated by OGD (4.08 ± 0.72-fold), while REOX caused downregulation (1.0 ± 0.04-fold). Within the same incubation conditions, stress factors did not have significant effects on every target (Table 2). Under 4 h NORMOX conditions, gene expression of ZO-1, occludin and integrin-α-1 was reduced by stress factors, whereas integrin-α-v was upregulated in comparison with untreated controls. OGD combined with stress factors affected mRNA levels of ZO-1, occluding, and VE-cadherin compared to OGD alone. After reoxygenation combined with stress factors gene expression of claudin-5, ZO-1, VE-cadherin, and integrin-α-1 was decreased in comparison with REOX alone. Subsequently, the data shown in Table 2 under REOX and REOX/stress factors were then used again in Table 3, but in this case the mRNA expression of selected targets was normalized to 24 h NORMOX (Table 3). The comparison in Table 2 enables an assessment of the gene expression over time, while Table 3 shows the mRNA levels only at 24 h point under various conditions. Claudin-5 gene expression did not change significantly after REOX with and without stress factor treatment. ZO-1 and VE-Cadherin consistently showed the same tendencies as claudin-5. The mRNA levels of occludin (1.35 ± 0.07-fold) and integrin-α-1 (1.32 ± 0.07-fold), however, were upregulated by REOX, but only without stress factors. As already mentioned above, in this experiment stress factor administration did not have significant effects on every target as well (Table 3). Integrin-α-v gene expression was induced by 24 h NORMOX in combination with stress factors (1.82 ± 0.3-fold) compared to 24 h NORMOX alone. No significant change of integrin-α-v mRNA level by stress factor administration under REOX conditions was observed in comparison with REOX alone. However, REOX conditions combined with stress factor administration led to a significant decrease of the expression of all other target genes compared to REOX alone. Within 24 h NORMOX conditions stress factors caused reduced gene expression of claudin-5, ZO-1, occludin, VE-cadherin, and integrin-α-1 as well. Interestingly, effects of stress factor administration under REOX conditions did not differ from those under 24 h NORMOX conditions. As already observed in immunofluorescence staining, the effects of stress factors under normoxia conditions evolved over time, especially in the case of claudin-5 (Tables 2,3). After 4 h, mRNA levels of claudin-5 and VE-cadherin were not changed, whereas after 24 h of stress factor administration gene expression of all targets was significantly decreased.

**Table 2:** Modulated target gene expression in cEND cells by stress factors under oxygen glucose deprivation (OGD) conditions and after subsequent reoxygenation (REOX) in comparison with 4 h of normoxia (4 h NORMOX).

	**Claudin-5**	**ZO-1**	**Occludin**	**VE-cadherin**	**Itg α 1**	**Itg α v**
4 h NORMOX	1	1	1	1	1	1
4 h NORMOX/stress factors	0.92 ± 0.05 n.s.	0.3 ± 0.02 ***	0.21 ± 0.02 ***	0.73 ± 0.07 n.s.	0.54 ± 0.02 ***	3.8 ± 0.6 *
OGD	0.49 ± 0.07 ***§	0.7 ± 0.08 *§	0.34 ± 0.06 ***§	1.56 ± 0.11 ***§	0.69 ± 0.05 n.s.§	4.08 ± 0.72 *§
OGD/stress factors	0.56 ± 0.11 **&	0.33 ± 0.03 ***$	0.08 ± 0.02 ***$	1.06 ± 0.07 n.s.$&	0.47 ± 0.07 ***	6.5 ± 1.08 ***
REOX	0.88 ± 0.07 n.s.	1.26 ± 0.06 n.s.	1.12 ± 0.07 n.s.	1.03 ± 0.03 n.s.	1.43 ± 0.12 **	± 0.04 n.s.
REOX/ stress factors	0.29 ± 0.06 ***#%	0.28 ± 0.1 ***#	0.26 ± 0.05 ***#	0.41 ± 0.06 ***#%μ	0.05 ± 0.06 ***#%μ	1.47 ± 0.05 n.s.μ

After total RNA-extraction from lysed cells, mRNA was quantified by real-time PCR. Fold expression vs. untreated control (4 h NORMOX), which was set 1. All values are the means (± SEM) of n = 10 independent experiments,*: statistically significant vs. 4 h or normoxia (4 h NORMOX) (*P < 0.05, **P < 0.01, ***P < 0.001. n.s, not significant), §, REOX statistically significant vs. OGD; $, OGD/stress factors vs. OGD; #, REOX/stress factors vs. REOX; &, OGD/stress factors vs. 4 h NORMOX/stress factors; %, REOX/ stress factors statistically significant vs. 4 h NORMOX/ stress factors; μ, REOX/stress factors statistically significant vs. OGD/stress factors.

**Table 3:** Modulated target gene expression in cEND cells by stress factors under oxygen glucose deprivation with subsequent reoxygenation (REOX) in comparison with 24 h of normoxia (24 h NORMOX).

	**Claudin-5**	**ZO-1**	**Occludin**	**VE-cadherin**	**Itg α 1**	**Itg α v**
24 h NORMOX	1	1	1	1	1	1
24 h NORMOX/stress factors	0.25 ± 0.05 ***	0.15 ± 0.05 ***	0.08 ± 0.01 ***	0.46 ± 0.05 ***	0.19 ± 0.05 ***	1.82 ± 0.3 **
REOX	0.87 ± 0.03 n.s.	1.12 ± 0.04 n.s.	1.35 ± 0.07 ***	1.05 ± 0.03 n.s.	1.32 ± 0.07 **	± 0.04 n.s.
REOX/stress factors	0.33 ± 0.06 ***§	0.24 ± 0.08 ***§	0.32 ± 0.06 ***§	0.32 ± 0.01 ***§	0.05 ± 0.01 ***§	1.51 ± 0.12 n.s.

After total RNA-extraction from lysed cells, mRNA was quantified by real-time PCR. Fold expression vs. untreated control (24 h NORMOX), which was set 1. Target gene expression was normalized to calnexin mRNA and shown as fold over untreated control, which was constantly set 1. All values are the means (± SEM) of n = 10 independent experiments, followed by statistical significance **P < 0.01, ***P < 0.001. n.s, not significant vs. 24 h NORMOX, § REOX/stress factors statistically significant vs. REOX, REOX/stress factors is not statistically significant vs. 24 h NORMOX/stress factors.

### Protein Expression in cEND Cells Is Changed by Elevated Catecholamine Levels and Inflammatory Mediators Under Oxygen Glucose Deprivation Conditions

**Figure 3:**
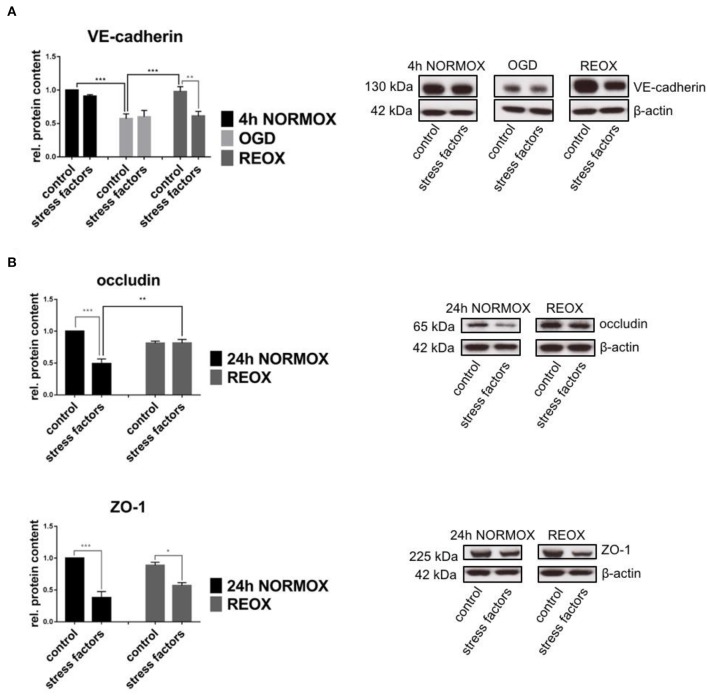
Protein expression in cEND cells is changed by elevated catecholamine levels and inflammatory mediators under oxygen glucose deprivation conditions. Cells were grown to confluence with subsequent differentiation. Stress factors were applied under different incubation conditions. Protein levels were examined by Western blot. Changed protein expression was normalized to β-actin. Changes of protein levels determined by densitometric analysis are expressed as fold over untreated control, which was set as 1. **(A)** VE-cadherin protein expression after stress factor administration under oxygen glucose deprivation conditions (OGD) and subsequent reoxygenation (REOX) in comparison with 4 h of normoxia (4 h NORMOX). Values are the means (± SEM) of *n* = 8 independent experiments. **(B)** Occludin and ZO-1 protein levels changed by stress factors after reoxygenation conditions (REOX) compared to 24 h of normoxia conditions (24 h NORMOX). Values are the means (± SEM) of *n* = 10 independent experiments. *P < 0.05, **P < 0.01, ***P < 0.001.

As described above, differentiated cells were treated with stress factors under selected incubation conditions. To study changes in protein levels, cEND cells were lysed, and analyzed by Western Blot. Initially, protein levels were determined after administration of stress factors under OGD as well as REOX conditions in comparison with 4 h NORMOX conditions (Figure 3A). VE-cadherin expression was compromised by OGD to 0.57 ± 0.07-fold of control, followed by an increase up to 0.98 ± 0.07-fold after reoxygenation. This was not observed in combination with stress factors. Compared to the untreated control under the same incubation conditions, stress factors significantly influenced the protein content only in REOX samples. Claudin-5, occluding, and ZO-1 protein levels (all not shown) showed the same tendencies as VE-cadherin, but without statistical significance. Finally, changed protein levels after REOX were compared to 24 h NORMOX conditions (Figure 3B). Treatment with stress factors in combination with REOX led to an increase in occludin up to 0.81 ± 0.03-fold of control compared to stress factor administration under 24 h NORMOX conditions (0.49 ± 0.07-fold). Expression of ZO-1 protein stayed unaffected by REOX conditions in comparison with 24 h NORMOX. Stress factors impaired significantly ZO-1 protein expression in both 24 NORMOX and REOX samples compared to untreated controls. Claudin-5 and VE-cadherin (both not shown) showed the same tendencies. Similar to gene expression, the effects of stress factors under NORMOX conditions were apparently time-dependent. All protein levels were significantly decreased after 24 h of stress factor administration, but not after 4 h exposure (Figures 3A,B).

## Discussion

The cardiovascular system is regulated by a central autonomic network consisting among others of the insular cortex, which is exposed to higher risk of cerebrovascular disease (10, 33). Damage in insular cortex has been associated with myocardial injury and TTS (10, 34). Based on clinical data, the simultaneous incidence of ischemic stroke and TTS has a poor outcome (1, 5, 11). Increased CAT and INF levels are one of the TTS characteristics (6). To date, the molecular mechanisms underlying these events are not fully elucidated. We hypothesized that the characteristic clinical constellation of elevated CAT and INF levels combined with OGD leads to an aggravated breakdown of the BBB, which subsequently contributes to severe ischemic stroke and poor clinical outcomes. In this study, we used an *in vitro* BBB model to study the effects of CAT/INF/OGD treatment. To our best knowledge, this is the first report examining BBB properties under such conditions.

Breakdown of the BBB is a key feature of neuroinflammatory conditions as encountered in multiple sclerosis (MS), meningitis, brain tumors, encephalitis, and cerebral ischemia, which inevitably leads to weakening of the vessels and predisposing them to leakage and rupture (35–37). BBB breakdown in frame of TTS could potentially be mediated by the effects of pro-inflammatory cytokines and CAT forming the basis for increased vulnerability to cerebrovascular dysfunction and stroke. In serum of acute phase TTS patients elevated levels of CAT such as dopamine, epinephrine, and norepinephrine and the pro-inflammatory cytokines TNFα, IL6, and IL10 have been reported (6, 38). In the pathology of ischemic stroke, TNFα and IL6 appear to be associated with BBB damage as seen on enhanced magnetic resonance imaging (MRI) scans (39). Using a rat model of experimental ischemia, it has been postulated that CAT are released in response to stress and ameliorate the ischemic brain damage (40). To address these observations at the molecular level, we decided to develop an *in vitro* pathology model of TTS based on our well-characterized murine brain endothelial cell line cEND (21). We previously validated this BBB model as suitable to study the barrier-compromising effects of pro-inflammatory stimuli and OGD, demonstrating that incubation of cEND monolayers with TNFα or OGD led to a down-regulation of TJ proteins and changes in pro-inflammatory cytokine expression (18, 20, 27, 41–43). Moreover, cEND express high levels of TJ, AJ, and ECM proteins and were used for permeability studies (41, 44, 45). In this study, we expand our characterization to the combined barrier-compromising effects of INF, CAT, and OGD conditions as partially encountered in acute phase TTS. We demonstrate compromised endothelial barrier function and reduced or altered junctional protein expression, resulting from incubation of cEND with (i) INF of TTS, (ii) with CAT elevated in TTS or, (iii) the combination of INF and CAT. As markers of BBB disruption we chose a barrier-integrity representing targets such the TJ and AJ components Cldn5, occludin, ZO-1, and VE-cadherin. As markers for interaction with ECM we chose Itg α1 and Itg αv, which were previously demonstrated to be altered in inflammatory processes (32). We observed an alteration in cellular morphology and cell number following incubation of cEND monolayers with TTS acute phase CAT/INF administered separately and simultaneously. Under normoxic conditions, morphology, monolayer integrity, cell number as well as TJ forming protein levels were more affected by incubation with CAT than INF. This was significantly mirrored at the expression level of analyzed marker proteins. Messenger RNA levels of ZO-1 and VE-cadherin were reduced by both treatments, while occludin was downregulated by INF, and claudin-5 by CAT. Expression levels of Itg α1 was reduced in response to INF/CAT treatment. Concomitantly, an upregulation of the αv of vitronectin was observed. Matching data supporting the important role of functional integrin-ECM binding have been demonstrated *in vivo*, while breakdown of the BBB has been observed in mice lacking selected integrins (46). In addition, altered expression patterns of specifically Itg α1 and Itg αv have been observed in MS lesions (47). The influence of altered integrin expression pattern on cellular differentiation has been described in integrin knock out mice and for epithelial and endothelial cells *in vitro* (32, 48, 49). Moreover, a role of integrins in epithelial barrier formation has been described in mammary epithelial cells (50). This supports the notion that a reduction of Itg α1 levels with concomitant increase in Itg αv levels can be considered a reliable marker of endothelial inflammation in TTS as well.

Increasing the permeability of the endothelial barrier caused by impaired cell-cell adhesion is believed to be an early event in ischemic stroke (51). Accordingly, increased barrier breakdown was observed under OGD with common TTS stressors, most notably under OGD/REOX conditions corresponding to ischemia-reperfusion injury. Monolayer morphology, monolayer integrity, and cell count were significantly affected in response to INF/CAT in OGD. CAT is known to induce apoptosis. CAT treatment of oligodendrocytes cultures induced apoptosis mediated by oxidative stress (52). TNFα induces apoptosis in human endothelial cells of various vascular beds (53, 54). A combination of all these factors escalated the effects on cEND and resulted in significant cell loss. Morphological changes were accompanied by decreased protein and mRNA levels of marker proteins. Interestingly, occludin, ZO-1, and Itg α1 were more affected by INF/CAT than claudin-5. These results indicate a susceptibility of ZO-1 and Itg α1 to INF/CAT/OGD, which are both key components of TJ complexes and cell matrix, respectively. The effect of the administered stress factors is time-dependent and does not have the same dimension for each target chosen. In addition, stress factors only partially amplify OGD effects. Possible intracellular pathways that may mediate the effects of supra-physiological CAT concentrations on cEND monolayers will have to be investigated in future studies. However, available literature information points toward a role for β-adrenergic stimulation (and cAMP mobilization) as an important barrier enhancing mechanism: (i) Chi et al. showed that a direct application of a beta-adrenergic receptor agonist to the brain parenchyma increased the permeability of the BBB, and that this effect could be prevented with a beta-adrenoceptor antagonist (55). (ii) Murphy and Johanson had proven the adrenergic-induced enhancement of brain barrier system permeability to small nonelectrolytes (56). (iii) A possible molecular pathway that could be involved is highlighted by the work of Ohara et al. (57). The beta 2-adrenergic receptor/cAMP pathway was shown to be involved in TNFα-stimulated ICAM-1 expression, which indicates the possible involvement of adrenergic mediation of BBB integrity, given (iiii) that the physiological role of ICAM-1 is to mediate neutrophil BBB transmigration (58). (iiiii) Neutrophils in turn mediate immune cell mediated communication between the immune system and the brain, which is specifically enhanced in systemic inflammatory insults that can exacerbate ongoing brain pathologies via immune cell trafficking (59).

An important limitation of the present study is that we only analyzed selected stress factors present in TTS *in vitro*. It is not clear, how the observed effects develop *in vivo* over time. *In vitro*, a stable concentration of stressors without preconditioning was used. In this model, we cannot determine whether increasing stress factors will affect the BBB in other ways over time. In addition, we used a simplified BBB *in vitro* model based on only one cell type, the brain microvascular endothelial cells, derived from mice. Other neurovascular unit cells such as pericytes and astrocytes are known to play an important role in maintaining the barrier *in vivo* and *in vitro* (60, 61). Differences in species could also play a role in our system (62). Therefore, studies with TTS stress factors will be carried out in future using a human *in vitro* BBB model in co-culture with astrocytes and pericytes.

## Conclusion

In summary, the present study provides the first evidence for the impairment of BBB by TTS stressors, CAT, and INF. In addition, the barrier-compromising effects of CAT and INF were highest during the reoxygenation phase, suggesting that TTS stressors along with other pathophysiological mechanisms, could contribute to secondary brain damage after stroke.

## Data Availability Statement

All datasets generated for this study are included in the article/supplementary material.

## Author Contributions

CI performed experiments and wrote the manuscript. CI, MB, SS, MN, and CF designed the experiments, analyzed, and interpreted the data. All authors critically revised the manuscript.

## Conflict of Interest

The authors declare that the research was conducted in the absence of any commercial or financial relationships that could be construed as a potential conflict of interest.
